# Cataract Surgery with or without Intraocular Lens Implantation in Pediatric Uveitis: A Systematic Review with Meta-Analyses

**DOI:** 10.1155/2021/5481609

**Published:** 2021-06-11

**Authors:** Diana Chabané Schmidt, Moug Al-Bakri, Asrin Rasul, Regitze Bangsgaard, Yousif Subhi, Daniella Bach-Holm, Line Kessel

**Affiliations:** ^1^Department of Ophthalmology, Rigshospitalet-Glostrup, Glostrup, Denmark; ^2^Department of Clinical Medicine, University of Copenhagen, Copenhagen, Denmark

## Abstract

**Purpose:**

To systematically review the results of comparative studies of modern cataract surgery in pediatric uveitis with or without intraocular lens (IOL) implantation and to perform comparative meta-analyses to compare visual acuity outcomes and complication rates.

**Methods:**

On 12 November 2020, we systematically searched the Cochrane Central, PubMed/MEDLINE, EMBASE, ClinicalTrials.gov, and all affiliated databases of the Web of Science. Two authors independently reviewed studies and extracted data. Studies were reviewed qualitatively in text and quantitatively with meta-analyses. Outcome measures were preoperative and postoperative best-corrected visual acuity (BCVA), inflammation control, and rates of postoperative complications.

**Results:**

Ten studies of 288 eyes were eligible for review of which the majority were eyes with juvenile idiopathic arthritis-associated uveitis. Summary estimates revealed that the BCVA was better in pseudophakic eyes vs. aphakic eyes (1-year postoperative: −0.23 logMAR, 95% CI: −0.43 to −0.03 logMAR, *P*=0.027; 5-year postoperative: −0.35 logMAR, 95% CI: −0.51 to −0.18 logMAR, *P*=0.000036). Pseudophakic eyes had more visual axis opacification (OR 6.76, 95% CI: 2.73 to 16.8, *P*=0.000036) and less hypotony (OR 0.19, 95% CI: 0.04 to 0.95, *P*=0.044).

**Conclusions:**

In modern era cataract surgery on eyes with pediatric uveitis with IOL implantation leads to satisfactory and superior visual outcomes and no differences in complication rates apart from an increased prevalence of visual axis opacification and a decreased prevalence of hypotony when compared to aphakia. However, limitations of the retrospective design and the presence of selection bias necessitate a careful interpretation.

## 1. Introduction

Pediatric uveitis is a challenging condition with an annual incidence of 4.3–6.9 per 100.000 children under the age of 16 years [1–3]. The condition often has an asymptomatic course and children tend to underreport visual changes resulting in advanced disease at the time of diagnosis [4, 5]. Complications such as cataract, ocular hypertension/glaucoma, amblyopia, cystoid macula edema (CME), posterior synechiae, band keratopathy, vasculitis, vitreous haze, and papillitis can be seen in case of delayed referral [6, 7]. These complications of pediatric uveitis lead to severe visual impairment in 18–38% of the patients [8–10]. Cataract is seen in up to 2/3 of patients with pediatric uveitis and is a complication related to chronic inflammation, prior surgical procedures (such as trabeculectomy or vitrectomy for retinal detachment), or prolonged treatment with glucocorticoids [5, 9, 11–13]. In these patients, it may be necessary to remove the cataract if it causes significant visual impairment, to prevent amblyopia, or to ensure adequate monitoring of the inflammation and the retina.

Cataract surgery in pediatric uveitis is technically challenging due to higher rates of ocular comorbidities, inflammatory sequelae, and structural abnormalities [14]. Intraocular lens (IOL) implantation in pediatric uveitis has been controversial and aphakia after cataract surgery has previously been practiced as a rule of thumb. Historically, early studies reported poor visual acuity after IOL implantation as well as a high rate of complications such as posterior synechiae, retrolental membranes, CME, secondary glaucoma, hypotony, and phthisis bulbi [15, 16]. This was ascribed to challenges in the surgical technique, increased ocular inflammation with IOL implantation, and lack of sufficient management of inflammation [17]. Recent technological advancements in the IOL design, biocompatible IOL materials, and modern surgical techniques, as well as immunomodulatory therapy, have improved inflammatory control pre- and postoperatively, all of which leads to better outcomes with IOL implantation according to more recent studies [18–21]. Despite the positive results reported in recent studies, IOL implantation remains controversial [22, 23].

The purpose of this study was to systematically review the results of comparative studies of modern cataract surgery in pediatric uveitis with or without IOL implantation and conduct meta-analyses to summarize and compare important outcomes.

## 2. Materials and Methods

This systematic review was conducted in accordance with the Preferred Reporting Items for Systematic Reviews and Meta-Analyses (PRISMA) [24]. For all aspects of this study, we followed the recommendations of the Cochrane Handbook [25]. Institutional review board approval was not relevant for systematic reviews according to Danish law.

### 2.1. Eligibility Criteria

We defined eligible studies as those fulfilling the following criteria.

 
*Population.* Studies of a pediatric population (individuals below 18 years of age) with any uveitis who undergo cataract surgery. We restricted to studies that only considered a pediatric population or studies that included such individuals as a subset of the study sample where data from such individuals could be extracted.
 
*Intervention*. Posterior chamber IOL implantation in the bag.
 
*Comparator*. Aphakia.
 
*Outcomes*. Short-term (1 year) and long-term (5 years) results of best-corrected visual acuity (BCVA) were defined as the primary outcome. Secondary outcomes were defined as specific incidence of the following within 5 years: anterior chamber inflammation, need for topical steroids, need for systemic immunosuppressive treatment, glaucoma (using the authors definition) or ocular hypertension, hypotony, need for resurgery for any reason, need for IOL explantation, visual axis opacification (posterior capsular opacification (PCO) and pupillary membrane formation), synechiae, phthisis bulbi, cystoid macular edema (CME), and retinal detachment.
 
*Study Types*. Eligible studies could be prospective or retrospective. We did not restrict based on randomization, blinding, or any other initiative to reduce bias. We included relevant abstracts, but not studies without original data or case reports. We did not restrict studies based on geography or journal. We only considered studies disseminated in English language. Since we want to focus on outcomes of modern cataract surgery, we only considered publications from year 2000 and onwards.

### 2.2. Information Sources, Search, and Study Selection

We searched the literature databases the Cochrane Central, PubMed/MEDLINE, EMBASE, Web of Science Core Collection, BIOSIS Previews, Current Contents Connect, Data Citation Index, Derwent Innovations Index, KCI-Korean Journal Database, Russian Science Citation Index, SciELO Citation Index, CINAHL, and ClinicalTrials.gov. The search was conducted on 12 November 2020. Details of the search strategy across databases are available as Supplementary file 1. One author (Y. S.) examined title and abstracts of all identified records, removed duplicates, and obviously irrelevant reports. Two authors (Y. S. and A. R.) independently screened remaining references in full text to evaluate eligibility of studies. Disagreements were discussed between the two authors and if consensus could not be reached, a third author (L. K.) would be invited for final decision. All reference lists were reviewed for identification of further relevant studies.

### 2.3. Data Extraction and Risk of Bias Assessment

We extracted data regarding study design, participant characteristics, and outcomes using predesigned data extraction forms. Two authors (Y. S. and D. C. S.) extracted all data independently. Based on our a priori knowledge of the literature, we anticipated nonrandomized comparative studies. Therefore, quality of eligible studies was assessed using the Risk of Bias in Nonrandomized Studies of Interventions (ROBINS-I) tool as recommended by Cochrane Methods [25, 26]. Two authors (Y. S. and M. A-B.) evaluated risk of bias independently. Disagreements between the authors were discussed and if consensus could not be reached, a third author (L. K.) would be invited for final decision.

### 2.4. Data Analysis and Synthesis

Eligible studies were described in text and tabulated for a qualitative synthesis. Due to the nonrandomized nature of available studies, we summarized and compared preoperative demographic and clinical characteristics of the intervention and the comparison group. All BCVA data were converted to logMAR for analyses [27]. For very low vision, we used the following conversion: no light perception = 2.9 logMAR, light perception = 2.6 logMAR, hand motion = 2.3 logMAR, and counting fingers = 1.9 logMAR [27]. For BCVA, we compared preoperative values as well as the postoperative results at short-term (1 year) and at long-term (5 years). Where no data was available specifically for 1 or 5 years, measures closest to these dates were used. All meta-analyses were performed using MetaXL 5.3 (EpiGear International, Sunrise Beach, QLD, Australia) for Microsoft Excel 2013 (Microsoft, Redmond, WA, USA). We used the random-effects model for our meta-analyses. Heterogeneity was assessed with Cochran's *Q* and quantified with I2 [28]. A Funnel plot was used to investigate for skewed results (risk of bias across studies) [29]. However, acknowledging the small number of studies potentially available, heterogeneity and risk of bias across studies were interpreted with caution. Sensitivity analyses were made to explore robustness of the estimates. All summary estimates are presented with 95% confidence intervals (CI) and *P* values. *P* values below 0.05 were interpreted as statistically significant.

## 3. Results

### 3.1. Study Selection

**Figure 1:**
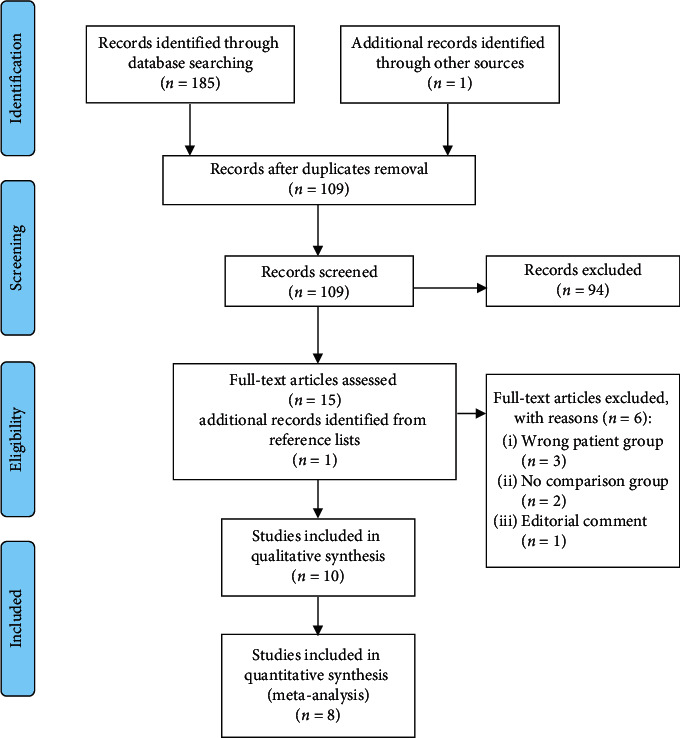
Flow diagram of study selection.

The literature search identified 185 records. Of these, 77 were duplicate records, 76 records were obviously irrelevant, and 18 records were not published in English language. One study known *a priori* to us was added to the reference list. The remaining 15 records were read in full text. One additional eligible study was identified by reviewing reference lists. Finally, 10 studies were eligible for the qualitative review and nine for quantitative synthesis (Figure 1).

### 3.2. Study Characteristics

The 10 studies collectively summarized data on 202 patients (Table 1). Three studies were only available as conference abstracts [30–32]. All were nonrandomized studies comparing groups obtained through retrospective chart reviews. Studies were from the USA (*n* = 4), Europe (*n* = 4), India (*n* = 1), and Israel (*n* = 1). Mean age of uveitis diagnosis ranged from 4 to 8 years. Mean age of cataract surgery ranged from 5 to 11 years. All studies had at least 1 year of follow-up and four studies had at least 5 years of follow-up.

Study populations were predominantly of eyes with juvenile idiopathic arthritis- (JIA-) associated uveitis (Table 2). Non-JIA-associated uveitis included Behçet's disease, herpes zoster virus uveitis, HLA-B27 associated uveitis, ocular tuberculosis, pars planitis, sarcoidosis, toxocariasis, Vogt-Koyanagi-Harada disease, and idiopathic uveitis (Table 2). Three studies reported that the uveitis was quiescent 3 months prior to surgery in all eyes [21, 35, 36], one study reported that the uveitis was inactive in 6 months prior to surgery [34], and one study reported absence of inflammation in 3 months prior to surgery except one eye with absence of inflammation for 2 months [33]. Three studies do not report on the degree of preoperative inflammation [30, 31, 37] and two studies operated all eyes despite active inflammation [16, 20].

Across the 10 studies, a total of 288 eyes underwent cataract surgery, of which 166 eyes had posterior chamber IOL implantation in the bag and 122 eyes were left aphakic. Four studies reported data on a very small number (3 or below) of aphakic eyes while no such small numbers were observed in the group of eyes with IOL implantation. Demographic and clinical factors differed in three studies [16, 21, 35] without any clear trend across studies (Table 3). In BenEzra and Cohen [16], the aphakic group differed by better preoperative BCVA [16]. In Quinones et al. [35], more cases of JIA-associated uveitis were in the left aphakic eyes [35]. Yangzes et al. [21] had more cases of panuveitis and poorer preoperative BCVA in left aphakic eyes [21]. None of the studies had a significant difference in age at cataract surgery between the study groups.

## 4. Results of Individual Studies and Risk of Bias within Studies

Artigas et al. [30] did not report visual acuity but found that IOL implantation leads to more frequent visits due to PCO and glaucoma development compared to aphakia [30]. The authors conclude that these visits should be taken into consideration when planning surgery [30]. Beal and Wang [31] found a nonsignificant trend towards better visual acuity in pseudophakic patients compared to the BCVA in aphakic patients and no differences were found in subsequent glaucoma development [31]. BenEzra and Cohen [16] described a practice where a choice of primary IOL implantation or aphakia was presented for cases with unilateral disease or young children with markedly unequal bilateral disease and the presence of dense cataract in one eye, whereas aphakia was the only presented option for children with bilateral disease and similar affection in both eyes [16]. They found that cataract surgery benefitted patients and improved visual acuity regardless of being pseudophakic or aphakic but that contact lenses were poorly tolerated especially among the young children [16]. Guindolet et al. [32] presented results of cataract surgery with either hydrophobic primary IOL implantation or aphakia [32]. Here, primary IOL implantation lead to good and prompt visual rehabilitation, but in comparison to aphakic patients, patients with IOL implantation had a higher postoperative oral corticosteroid use [32]. Kemp et al. [33] found cataract surgery with primary IOL implantation to yield satisfactory outcomes as all eyes achieved visual acuity of 20/30 or better, and no differences were found in use of medications after surgery between pseudophakic and aphakic patients [33]. Kotaniemi and Penttilä [20] investigated outcomes after change in practice from aphakia to primary IOL implantation [20]. Primary IOL implantation improved visual acuity and visual acuity of ≥0.5 Snellen was achieved in 64% of eyes with IOL [20]. In this study, comparison could only be made to the few patients with contralateral eye who had cataract surgery with aphakia prior to the implementation of new practice [20]. O'Rourke et al. [34] found that IOL implantation leads to excellent visual acuity (defined as >6/9.5 Snellen) but that comorbidities such as glaucoma, band keratopathy, and CME all required a tight postoperative care and that 80% of eyes had uveitis flare-ups [34]. They left one eye aphakic due to preexisting advanced uveitic glaucoma and difficulties in satisfactory immunosuppression; this eye did not improve in BCVA [34]. Sijssens et al. [36] compared cataract surgery with aphakia to primary IOL implantation, in which the latter had presurgical history of a higher rate of glaucoma history, trabeculectomy, and treatment with methotrexate [36]. In this comparative study, the authors found that the BCVA improved significantly more in the pseudophakic eyes than in the aphakic eyes [36]. Yangzes et al. [21] found that cataract surgery improved vision in eyes regardless of IOL implantation or not, the rate of glaucoma development was comparable between the groups, but PCO leads to more secondary procedures in the pseudophakic eyes [21]. Taken together, studies found that cataract surgery, regardless of IOL implantation, generally improved vision. Nearly all studies specifically highlighted the need for intensive immunosuppressive treatment and control of uveitis after cataract surgery, but it was unclear whether it was a question of sustaining preexisting regimen or based on a change in the need for controlling the uveitis [16, 20, 21, 32–36].

Risk of bias assessment was challenged in three studies since we only had access to conference abstracts with limited information [30–32]. Remaining studies had moderate-to-serious risk of bias (Table 4), in which the key source of bias was the baseline confounding from the fact that the allocation to either IOL implantation or aphakia across studies was based on the individual surgeon's estimation of whether or not pseudophakia or aphakia would benefit the patient best.

### 4.1. Synthesis of Results in Meta-Analyses and Risk of Bias across Studies

Nine studies provided eligible and comparable data for the meta-analyses [16, 20, 21, 30, 32–36]. These studies collectively summarized data on 256 eyes: 153 eyes underwent IOL implantation and 103 eyes were aphakic.

Primary outcomes: short-term and long-term results on best-corrected visual acuity.

Eight studies provided relevant data for the primary outcome [16, 20, 21, 32–36]. O'Rourke et al. [34] provided only data on a single eye with aphakia, which leads to SD = 0, and therefore this study was ineligible for the meta-analysis for analytical reasons [34]. Thus, seven studies were included for the meta-analyses of the primary outcome [16, 20, 21, 32, 33, 35, 36].

**Figure 2:**
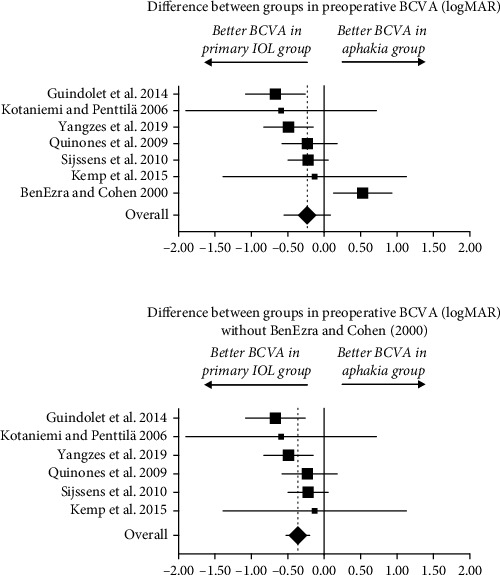
Forest plot of the differences between groups in the preoperative best-corrected visual acuity (BCVA). Top: Primary analysis with all eligible studies. In this analysis, BenEzra and Cohen [16] introduced a significant heterogeneity relative to the other studies. Bottom: Analyses were repeated after excluding BenEzra and Cohen [16], which significantly reduced heterogeneity. Summary estimates are weighted mean difference (WMD) in logMAR.

For preoperative BCVA, the random-effects pooled weighted mean difference between those with primary IOL implantation and aphakia was −0.23 logMAR (95% CI: −0.55 to 0.08 logMAR, *P*=0.15), i.e., the preoperative BCVA did not differ significantly between the two populations (Figure 2). Cochran's *Q* of 20.95 and *I*^2^ of 71% were both indicative of a large heterogeneity across studies, and the Funnel plot appeared symmetrical apart from the outlier from BenEzra and Cohen [16] (Supplementary file 2). Our sensitivity analysis revealed that excluding BenEzra and Cohen [16], which unlike the other studies had significantly better preoperative BCVA in the aphakia group, would completely change the conclusions from our initial calculations. Excluding BenEzra and Cohen [16] leads to a random-effects pooled weighted mean difference of −0.36 logMAR (−0.52 to −0.20 logMAR, *P*=0.000014); i.e., the preoperative BCVA was significantly better in eyes planned for primary IOL implantation compared to those planned for aphakia (Figure 2). This analysis had much less heterogeneity across studies: Cochran's *Q* = 4.59 and *I*^2^ = 0%. A separate sensitivity analysis of this subanalysis showed strong robustness of the analysis as excluding studies in turn did not significantly change the size (range −0.30 to −0.44 logMAR), the direction (all in favor of primary IOL implantation), or the statistical significance of the findings (Supplementary file 2).

**Figure 3:**
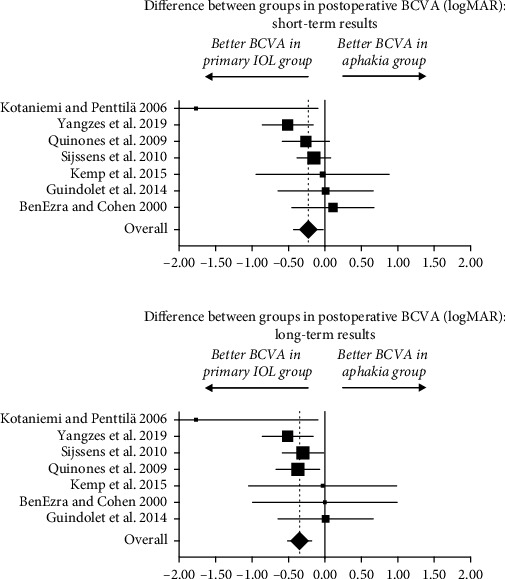
Forest plot of the differences between groups in postoperative short-term (1 year, top) and long-term (5 years, bottom) outcomes in the best-corrected visual acuity (BCVA). Summary estimates are weighted mean difference (WMD) in logMAR. To allow for easier interpretation of the overall study results, we refrained from adjusting figure to the study outcomes from Kotaniemi and Penttilä [20] which were subject to very large confidence intervals (−3.44 to −0.09 and −3.44 to −0.09, respectively, for short-term and long-term results).

For short-term results on postoperative BCVA, the random-effects pooled weighted mean difference between primary IOL implantation and aphakia was −0.23 logMAR (95% CI: −0.43 to −0.03 logMAR, *P*=0.027); i.e., primary IOL implantation leads to significantly better BCVA on the short-term (Figure 3). A Cochran's *Q* of 8.34 and *I*^2^ of 28% were indicative of small heterogeneity across studies. The Funnel plot appeared symmetrical (Supplementary file 3). Sensitivity analysis revealed that excluding either Quinones et al. [35], Sijssens et al. [36], or Yangzes et al. [21] would lead to loss of the statistical significance of the findings; hence short-term differences did not show robustness in the sensitivity analysis (Supplementary file 3).

For long-term results on postoperative BCVA, the random-effects pooled weighted mean difference between primary IOL implantation and aphakia was −0.35 logMAR (95% CI: −0.51 to −0.18 logMAR, *P*=0.000036); i.e., primary IOL implantation leads to significantly better BCVA on the long-term (Figure 3). A Cochran's *Q* of 5.74 and I^2^ of 0% were indicative of a small-to-none heterogeneity across studies. The Funnel plot appeared symmetrical (Supplementary file 4). Sensitivity analysis demonstrated robustness of the findings as excluding studies in turn did not significantly change the size (range −0.30 to −0.38 logMAR), the direction (all in favor of primary IOL implantation), or the statistical significance of the findings (Supplementary file 4).

Secondary outcomes: presence of inflammation (anterior chamber inflammation and cystoid macular edema) and the need for immunosuppression (topical steroids and systemic immunosuppressive treatment).

BenEzra and Cohen [16], Kemp et al. [33], and O'Rourke et al. [34] reported postoperative anterior chamber inflammation in terms of uveitis flares [16, 33, 34]. These outcomes were not reported sufficiently homogenous for a meaningful meta-analysis. In the BenEzra and Cohen study [16] two pseudophakic eyes (out of 10 eyes) and one aphakic eye (out of 10 eyes) experienced chronic intraocular inflammation after surgery [16]. In the Kemp et al. study [33], five pseudophakic eyes (out of six eyes) and none of the three aphakic eyes experienced uveitis flares [33]. In the O'Rourke et al. study [34], the only aphakic eye had three flare episodes, while the remaining nine pseudophakic eyes had three flare episodes in two eyes, in six eyes a single flare episode, and in two eyes no flare episodes [34].

Quinones et al. [35] reported anterior chamber cells in a grading system (<1+, 1+, 2+, >2+) during the postoperative follow-up period [35]. At final visit, eight pseudophakic eyes (62%) and 23 aphakic eyes (82%) had <1+ anterior chamber cells, which was not statistically significant [35].

Postoperative CME during the follow-up period in specific study groups was reported in five studies [16, 20, 21, 32, 36]. The random-effects risk estimate for postoperative CME between IOL implantation and aphakia was OR 0.70 (95% CI: 0.15 to 3.29, *P*=0.65) (Supplementary file 5), i.e., no significant difference in risk of postoperative CME between IOL implantation and aphakia. The Funnel plot appeared symmetrical and the sensitivity analysis demonstrated robustness of the findings (Supplementary file 5).

Kemp et al. [33], Kotaniemi and Penttilä [20], O'Rourke et al. [34], and Yangzes et al. [21] reported on the postoperative need for topical steroids and systemic immunosuppressive treatment [20, 21, 33, 34]. These outcomes were not reported sufficiently homogenous for a meaningful meta-analysis. Kemp et al. [33] reported that, postoperatively, all patients continued their preoperative immunomodulatory medications, which were different combinations of systemic prednisone, methotrexate, infliximab, adalimumab, and topical prednisolone acetate 1% [33]. Kotaniemi and Penttilä [20] reported that, at the end of follow-up (3.3 ± 3.2 years), topical corticosteroid treatment was ongoing in 33 (92%) pseudophakic eyes and 3 (100%) aphakic eyes [20]. Here, systemic immunomodulatory medications were either single treatment or a combination treatment of the following: prednisolone (17 patients), methotrexate (15 patients), and cyclosporine A (14 patients); in 9 patients, infliximab or etanercept were introduced but withdrawn in two patients due to inefficacy or allergy [20]. Further, the authors also tried other disease-modifying antirheumatic drugs (sulfasalazine, leflunomide, azathioprine, hydroxychloroquine, and chlorambucil) [20]. For all these systemic immunomodulatory medications, Kotaniemi and Penttilä [20] did not provide comparative data on pseudophakic vs. aphakic eyes [20]. O'Rourke et al. [34] reported that eight cases of uveitis flare-ups were managed with augmented topical treatment in three cases, dexamethasone intravitreal implant in one case, and Adalimumab in four cases of which Mycophenolate mofetil was added in two [34]. This study did not specify how the immunomodulatory treatments were distributed in pseudophakic vs. aphakic eyes [34]. Yangzes et al. [21] reported that systemic prednisolone was given in all cases in the postoperative period, that 23 patients (62%) received additional immunosuppressive treatment (methotrexate in 6, azathioprine in 7, and combination of methotrexate and azathioprine in 10), and that four eyes (8%) received dexamethasone intravitreal implant [21]. However, whether or not some of these medications were reduced or intensified during the follow-up period was not described [21].

### 4.2. Secondary Outcomes: Risk of Ocular Hypertension and Glaucoma

Ocular hypertension as a separate diagnosis was reported in two studies with slightly different definitions not sufficiently homogenous for inclusion in a meaningful meta-analysis [16, 32]. BenEzra and Cohen [16] reported that four of 10 aphakic eyes needed treatment to control intraocular pressure, whereas the pseudophakic group with 10 eyes had one case with uncontrollable intraocular pressure and development of intractable glaucoma [16]. Guindolet et al. [32] reported that four of 14 pseudophakic eyes had secondary ocular hypertension and none among the six aphakic eyes [32].

Six studies reported on presence of glaucoma [16, 20, 21, 30, 34, 36]. Some of these studies also counted cases of glaucoma prior to cataract surgery. The random-effects risk estimate for glaucoma between IOL implantation and aphakia was OR 1.52 (95% CI: 0.73 to 3.17, *P*=0.26) (Supplementary file 6), i.e., no significant difference in risk of glaucoma between IOL implantation and aphakia. The Funnel plot appeared symmetrical and the sensitivity analysis demonstrated robustness of the findings (Supplementary file 6).

### 4.3. Secondary Outcome: Visual Axis Opacification

Seven studies reported on the incidence of postoperative visual axis opacification, e.g., PCO or pupillary membrane formation [16, 20, 21, 30, 32–34]. Across all studies, any visual axis opacification was much more prevalent in the pseudophakic group [16, 20, 21, 30, 32–34]. The random-effects risk estimate for visual axis opacification between IOL implantation and aphakia was OR 6.76 (95% CI: 2.73 to 16.8, *P*=0.000037 (Supplementary file 7); i.e., IOL implantation leads to significantly higher risk of visual axis opacification. The Funnel plot appeared symmetrical and the sensitivity analysis demonstrated robustness of the findings (Supplementary file 7).

Secondary outcomes: risk of hypotony, synechiae, retinal detachment, and phthisis bulbi.

Four studies reported on the incidence of postoperative hypotony [16, 21, 34, 36]. The random-effects risk estimate for hypotony between IOL implantation and aphakia was OR 0.19 (95% CI: 0.04 to 0.95, *P*=0.044) (Supplementary file 8); i.e., IOL implantation leads to significantly lower risk of hypotony. We refrained from interpreting the Funnel plot or the sensitivity analysis due to the low number of studies in analysis (<5) (Supplementary file 8).

Postoperative posterior synechia was reported in three studies [16, 32, 33]. The random-effects risk estimate for posterior synechia between IOL implantation and aphakia was OR 3.70 (95% CI: 0.44 to 31.11, *P*=0.023) (Supplementary file 9), i.e., no significant difference in risk of posterior synechia between IOL implantation and aphakia. We refrained from interpreting the Funnel plot or the sensitivity analysis due to the low number of studies in analysis (<5) (Supplementary file 9).

Postoperative retinal detachment was reported in five studies [16, 20, 21, 32, 34]. The random-effects risk estimate for retinal detachment between IOL implantation and aphakia was OR 0.79 (95% CI: 0.18 to 3.57, *P*=0.76) (Supplementary file 10), i.e., no significant difference in risk of retinal detachment between IOL implantation and aphakia. The Funnel plot appeared symmetrical and the sensitivity analysis demonstrated robustness of the findings (Supplementary file 10).

Postoperative development of phthisis bulbi was only reported by Sijssens et al. [36]. Here, the authors reported one case among 19 aphakic eyes and no cases among the 29 pseudophakic eyes, which did not differ significantly (OR 0.21, 95% CI: 0.0081 to 5.41, *P*=0.35).

Secondary outcomes: risk of intraocular lens explantation or resurgery for any reason.

None of the 10 studies with 166 pseudophakic eyes reported need for lens explantation [16, 20, 21, 30–36]. A substantial number of both pseudophakic and aphakic eyes had resurgery, primarily because of glaucoma, but also due to visual axis opacification, band keratopathy, retinal detachment, and vitrectomy to manage chronic inflammation [16, 20, 21, 30–36]. It was not possible to extract data on number of eyes that had any resurgery (or eyes that did not have any resurgery) as data were not reported.

## 5. Discussion

This systematic review summarizes the evidence on modern cataract surgery in eyes with pediatric uveitis with either primary IOL implantation or aphakia. All ten studies eligible for review were retrospective chart reviews without randomization of eyes or patients, and further, several studies provided qualitative or quantitative evidence of selection bias. These limitations and the strong presence of selection bias must be kept in mind when interpreting the results of individual studies and our summary estimates. However, it is also important to realize that the evidence and estimates in this review are the best evidence the literature can present at this point.

**Figure 4:**
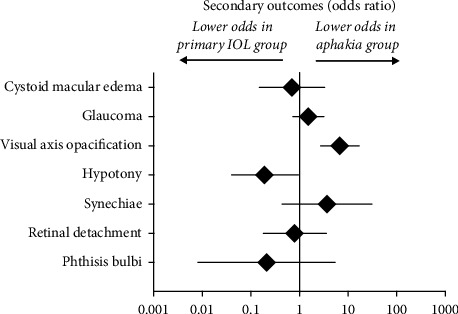
Overview of the secondary outcome meta-analyses. Summary estimates are odds ratio (OR). Significant differences between groups were visual axis opacification (OR 6.76, 95% CI: 2.73 to 16.8, *P*=0.000037, i.e., more likely in those with primary IOL implantation group/less likely in aphakia) and hypotony (OR 0.19, 95% CI: 0.04 to 0.95, *P*=0.044, i.e., less likely in those with primary IOL implantation group/more likely in aphakia).

Our meta-analyses revealed that the visual acuity was better in the IOL group one and five years after cataract surgery. Complications after cataract surgery in pediatric uveitis were included as secondary outcomes for the meta-analyses and a summary of these is presented in Figure 4. Compared to aphakia, statistically significant differences were only obtained in pseudophakia for higher rate of visual axis opacification and fewer cases of hypotony.

Included studies reported on different subtypes of pediatric uveitis. The various types of pediatric uveitis do not react to cataract surgery equally. JIA-associated uveitis is known to have a more severe manifestation of uveitis and a more complicated postoperative disease course than other types of uveitis [15, 16, 38]. Therefore, many surgeons may choose to leave eyes with JIA-associated uveitis aphakic. Quinones et al. [35] reported significantly more cases of JIA-associated uveitis in the aphakia group [35]. Similar considerations may underlie the decisions made in the study by Yangzes et al. [21], where the aphakia group had significantly more cases of panuveitis [21]. These studies highlight the selection bias that may influence our results. However, our review also includes data from a significant number of eyes with JIA-associated uveitis that underwent primary IOL implantation. In fact, more than half of the eyes (165 eyes out of 288) in this review had JIA-associated uveitis, and therefore it can be argued that primary IOL implantation can be an option for eyes with JIA-association uveitis but that randomized studies are warranted to determine the comparative efficacy and the primary choice of treatment.

Preoperative control of inflammation is generally recommended, and many prefer a practice of ≥3 months of quiescence before surgery to prevent complications and achieve the best possible visual acuity [37, 39, 40]. Five studies reported adequate preoperative immunosuppressive treatment [21, 33–36], three studies did not report if the eyes had been quiescent prior to surgery [30, 31, 41], and two studies reported that surgery was performed despite of inflammation [16, 20]. Considering that preoperative inflammation control impacts postoperative outcomes and that preoperative inflammation control was subject to a certain heterogeneity, the results of this review should be interpreted with caution. It has been feared that implanting a foreign body, an IOL, during surgery may trigger an immune response and influence the postoperative need for anti-inflammatory treatment. Most studies did not describe the pre- or postoperative immunosuppressive treatment in detail. Guindolet et al. [32] reported a higher postoperative corticosteroid use after IOL implantation [32], while Kemp et al. [33] did not find any difference in medication between IOL implantation and aphakia [33].

A multicenter study from Alió et al. [30] with 140 eyes compared implantation of different types of intraocular lens material in adult patients with uveitis [42]. They found that eyes with acrylic lenses had the lowest levels of postoperative inflammation in the first month and that heparin-coated PMMA lenses had the lowest incidence of uveitis relapses [42]. Silicone lenses had the highest rate of posterior capsular opacification and the highest rate of uveitis relapses [42]. Papaliodis et al. [43] found that implantation of acrylic lenses leads to less inflammation, fewer cases of PCO and CME, and better visual acuity when compared to heparin-coated PMMA, PMMA, or silicone lenses in a study with 36 eyes [43]. Studies in our review employed mainly acrylic or PMMA lenses, which may contribute to an explanation of the satisfactory clinical outcomes.

Not all children may be able to tolerate contact lenses after surgery and contact lens use concomitant with topical steroids to control inflammation may be problematic [16]. Aphakia spectacles can be impractical due to narrowing of the visual field and in case of unilateral cataract result in aniseikonia that affects stereopsis [44]. Therefore, a strong argument for choosing IOL implantation over aphakia is the easier optical rehabilitation.

## 6. Conclusion

Taken together, we conclude that in modern era cataract surgery of eyes with pediatric uveitis with IOL implantation leads to satisfactory and superior visual outcomes and no differences in complication rates apart from an increased prevalence of visual axis opacification and a decreased prevalence of hypotony when compared to aphakia. However, these results are subject to a certain degree of selection bias. Based on the current evidence and under careful patient selection and adequate pre- and postoperative inflammatory control, we consider IOL implantation to be a reasonable alternative to aphakia in pediatric uveitis. It must be stressed that randomized studies are needed to fully conclude which option should be considered superior or first line of therapy.

## Figures and Tables

**Table 1:** Characteristics of the included studies.

Reference	Study design	Patients and eyes, N	Country	Age at uveitis diagnosis, years	Age at cataract surgery, years	Females, (%)	Follow-up after cataract surgery, years
Artigas et al. [30]	Retrospective chart review	7 patients, 11 eyes	USA	N/A	7.5 ± 2.5	57%	5.8 ± 4.0

Beal and Wang [31]	Retrospective chart review	25 patients, 32 eyes	USA	N/A	N/A	N/A	4.0

BenEzra and Cohen [31]	Retrospective chart review	17 patients, 20 eyes	Israel	5.7 ± 3.8	9.1 ± 4.6	71%	5.0

Guindolet et al. [32]	Retrospective chart review	16 patients, 20 eyes	France	N/A	7.9 ± 2.8	N/A	3.0

Kemp et al. [33]	Retrospective chart review	7 patients, 9 eyes	USA	4.4 ± 1.8	5.4 ± 2.1	57%	1.6 ± 0.8

Kotaniemi and Penttilä [20]	Retrospective chart review	25 patients, 39 eyes	Finland	6.8 ± 5.8	11.3	84%	3.3

O'Rourke et al. [34]	Retrospective chart review	7 patients, 10 eyes	Ireland	7.7 ± 2.2	N/A	57%	7.4 ± 2.7

Quinones et al. [35]	Retrospective chart review	34 patients, 41 eyes	USA	6.7 ± 3.0	9.8 ± 3.3	71%	4.1 ± 3.9

Sijssens et al. [36]	Retrospective chart review	29 patients, 48 eyes	The Netherlands	4.2 ± 1.6	7.1 ± 2.5	62%	7

Yangzes et al. [21]	Retrospective chart review	37 patients, 58 eyes	India	N/A	10.5 ± 5.4	68%	3.7 ± 7.2

Data are presented in mean ± standard deviation where possible. IOL = intraocular lens; N/*A* = not available; USA = United States of America.

**Table 2:** Distribution of uveitis subtypes among eligible patients for this review.

Reference	Uveitis subtypes
Artigas et al. [30]	JIA-associated uveitis (11 eyes)

Beal and Wang [31]	Any uveitis (32 eyes)

BenEzra and Cohen [31]	JIA-associated (9 eyes) and non-JIA-associated uveitis (11 eyes)

Guindolet et al. [32]	JIA-associated (9 eyes) and non-JIA-associated uveitis (11 eyes)

Kemp et al. [33]	JIA-associated (7 eyes) uveitis, juvenile xanthogranulomatosis (1 eye), and idiopathic uveitis (1 eye)

Kotaniemi and Penttilä [20]	JIA-associated uveitis (39 eyes)

O'Rourke et al. [34]	Idiopathic uveitis (5 eyes), JIA-associated uveitis (2 eyes), ocular tuberculosis (2 eyes), and HLA-B27 associated uveitis (1 eye)

Quinones et al. [35]	JIA-associated uveitis (21 eyes), pars planitis (7 eyes), other uveitis (6 eyes; idiopathic, HZV-associated, sarcoid panuveitis)

Sijssens et al. [36]	JIA-associated uveitis (48 eyes)

Yangzes et al. [21]	JIA-associated uveitis (19 eyes), ocular tuberculosis (8 eyes), idiopathic uveitis (4 eyes), Behçet's disease (2 eyes), VKH disease (2 eyes), HLA-B27 associated uveitis (1 eye), and toxocariasis (1 eye)

HLA = human leukocyte antigen; HZV = herpes zoster virus; JIA = juvenile idiopathic arthritis; VKH = Vogt-Koyanagi-Harada.

**Table 3:** Demographic and clinical comparison of study groups.

Reference	IOL						No IOL					Significant differences
	Eyes, N	Age at surgery, years	Females, (%)	Uveitis subtypes	Pre-op BCVA, logMAR	Type of IOL	Eyes, N	Age at surgery, years	Females, (%)	Uveitis subtypes	Pre-op BCVA, logMAR	
Artigas et al. [30]	9	N/A	N/A	9 JIA	N/A	Alcon Acrysof	2	N/A	N/A	2 JIA	N/A	N/A

Beal and Wang [31]	13	N/A	N/A	N/A	N/A	N/A	19	N/A	N/A	N/A	N/A	N/A

BenEzra and Cohen [16]	10	9.2 ± 4.5	70%	5 JIA, 5 idiopathic	2.8 ± 0.1	3M style 925 (3 eyes) and Allergan PC26 TB (7 eyes)	10	10.3 ± 5.1	71%	3 JIA, 3 idiopathic, 1 *Toxocara*	2.3 ± 0.6	Better pre-op BCVA in the aphakia group

Guindolet et al. [32]	14	8.6 ± 7.9	N/A	N/A	1.1 ± 0.5	Hydrophobic acrylic lens	6	6.1 ± 4.5	N/A	N/A	1.8 ± 0.4	No

Kemp et al. [33]	6	6.8 ± 1.7	50%	5 JIA, 1 idiopathic	1.8 ± 1.0	Alcon MA50BM (8 eyes) or SA60AT (1 eye)	3	8.7 ± 6.4	67%	2 JIA, 1 xanthogranulomatosis	1.9 ± 0.8	No

Kotaniemi and Penttilä [20]	36	N/A	86%	36 JIA	1.0 ± 0.6	Hydrophobic acrylic (25 eyes) or PMMA (11 eyes) lens	3	N/A	33%	3 JIA	1.6 ± 1.1	No

O'Rourke et al. [34]	9	N/A	56%	5 idiopathic, 2 ocular tuberculosis, 1 JIA, 1 HLA-B27	0.7 ± 0.3	N/A	1	N/A	100%	1 JIA	1.0	No

Quinones et al. [35]	13	11.4 ± 4.4	N/A	7 idiopathic, 4 JIA, 2 other	0.9 ± 0.5	PMMA lens	28	9.4 ± 3.9	N/A	23 JIA, 5 idiopathic	1.1 ± 0.5	More JIA in the aphakia group

Sijssens et al. [36]	29	6.3 ± 2.0	72%	29 JIA	1.0 ± 0.5	Hydrophobic acrylic or PMMA lens	19	7.6 ± 2.7	72%	19 JIA	1.2 ± 0.5	No

Yangzes et al. [21]	27	10.9 ± 4.1	N/A	18 anterior, 6 intermediate, 3 panuveitis, 0 posterior	0.8 ± 0.7	Hydrophobic acrylic or PMMA lens	31	7.8 ± 5.1	N/A	12 anterior, 7 intermediate, 11 panuveitis, 1 posterior	1.3 ± 0.6	More panuveitis and worse pre-op BCVA in the aphakia group

Data are presented in mean ± standard deviation. Demographic data here are presented per eye where possible. Data were transformed to mean ± standard deviation where possible. For BCVA, we converted reported values to logMAR for better comparability within study and across studies. For logMAR conversion, we used the following for extreme low vision values: counting fingers = +2.3, hand motion = +2.6, light perception = +2.9, no light perception = +3.2. IOL = intraocular lens; N/*A* = not available; PMMA = polymethyl methacrylate; USA = United States of America.

**Table 4:** Risk of bias assessment for each study using the Risk of Bias in Nonrandomized Studies of Interventions (ROBINS-I) tool.

Reference	Bias due to confounding	Bias due to selection of participants	Bias due to classification of interventions	Bias due to deviations from intended interventions	Bias due to missing data	Bias in measurement of outcomes	Bias in selection of the reported results	Overall bias
Artigas et al. [30]	Unclear	Unclear	Unclear	Unclear	Low	Low	Unclear	Unclear

Beal and Wang [31]	Unclear	Unclear	Unclear	Unclear	Low	Low	Unclear	Unclear

BenEzra and Cohen [16]	Serious	Low	Low	Low	Low	Low	Moderate	Serious

Guindolet et al. [32]	Unclear	Unclear	Unclear	Unclear	Low	Low	Unclear	Unclear

Kemp et al. [33]	Unclear	Low	Low	Low	Low	Low	Moderate	Moderate

Kotaniemi and Penttilä [20]	Serious	Low	Low	Low	Low	Low	Moderate	Serious

O'Rourke et al. [34]	Serious	Low	Low	Low	Low	Low	Moderate	Serious

Quinones et al. [35]	Serious	Moderate	Moderate	Moderate	Low	Low	Moderate	Serious

Sijssens et al. [36]	Serious	Low	Low	Moderate	Low	Low	Low	Serious

Yangzes et al. [21]	Serious	Moderate	Moderate	Moderate	Low	Low	Moderate	Serious

Three studies [30–32] were conference abstracts, and risk of bias assessment of these studies was challenged by the limited insight obtainable from these abstracts.

## Data Availability

All data are included in this paper and its supplementary files.

## Supplementary Materials

Supplementary Materials
*Supplementary file 1*. Details of the literature search across different databases. *Supplementary file 2*. Details of the meta-analysis on the preoperative best-corrected visual acuity, including Funnel plot and sensitivity an1alysis. Supplementary file 3. Details of the meta-analysis on the postoperative short-term best-corrected visual acuity, including Funnel plot and sensitivity analysis. *Supplementary file 4*. Details of the meta-analysis on the postoperative long-term best-corrected visual acuity, including Funnel plot and sensitivity analysis. *Supplementary file 5*. Details of the meta-analysis on the postoperative cystoid macular edema, including Funnel plot and sensitivity analysis. *Supplementary file 6.* Details of the meta-analysis on the postoperative glaucoma, including Funnel plot and sensitivity analysis. *Supplementary file 7*. Details of the meta-analysis on the postoperative visual axis opacification, including Funnel plot and sensitivity analysis. *Supplementary file 8*. Details of the meta-analysis on the postoperative hypotony, including Funnel plot and sensitivity analysis. *Supplementary file 9*. Details of the meta-analysis on the postoperative posterior synechia, including Funnel plot and sensitivity analysis. *Supplementary file 10.* Details of the meta-analysis on the postoperative retinal detachment, including Funnel plot and sensitivity analysis.
