# Supplementary material for: Cataract Surgery with or without Intraocular Lens Implantation in Pediatric Uveitis: A Systematic Review with Meta-Analyses
Source: J Ophthalmol. 2021 Jun 11;2021:5481609. doi: 10.1155/2021/5481609 (PMC8213487; doi:10.1155/2021/5481609)
Supplement: Supplementary Materials — Supplementary file 1. Details of the literature search across different databases. Supplementary file 2. Details of the meta-analysis on the preoperative best-corrected visual acuity, including Funnel plot and sensitivity an1alysis. Supplementary file 3. Details of the meta-analysis on the postoperative short-term best-corrected visual acuity, including Funnel plot and sensitivity analysis. Supplementary file 4. Details of the meta-analysis on the postoperative long-term best-corrected visual acuity, including Funnel plot and sensitivity analysis. Supplementary file 5. Details of the meta-analysis on the postoperative cystoid macular edema, including Funnel plot and sensitivity analysis. Supplementary file 6. Details of the meta-analysis on the postoperative glaucoma, including Funnel plot and sensitivity analysis. Supplementary file 7. Details of the meta-analysis on the postoperative visual axis opacification, including Funnel plot and sensitivity analysis. Supplementary file 8. Details of the meta-analysis on the postoperative hypotony, including Funnel plot and sensitivity analysis. Supplementary file 9. Details of the meta-analysis on the postoperative posterior synechia, including Funnel plot and sensitivity analysis. Supplementary file 10. Details of the meta-analysis on the postoperative retinal detachment, including Funnel plot and sensitivity analysis. [file 5481609.f1.pdf]

Supplementary file 1. Details of the literature search across different databases.

PubMed:
History and Search Details

Search	Actions	Details	Query	Results	Time
#1			Search: **(juvenile OR paediatic* OR pediatric* OR child* OR infant* OR "Child"[Mesh] OR "Pediatrics"[Mesh] OR "Infant"[Mesh]) AND (uveitis OR uveitic OR uveit* OR "Uveitis"[Mesh]) AND ("intraocular lens" OR IOL OR "Lenses, Intraocular"[Mesh]) AND (aphakic OR aphakia OR aphak* OR "Aphakia"[Mesh])** Sort by: **Most Recent** ("juvenile"[All Fields] OR "juvenile s"[All Fields] OR "juveniles"[All Fields] OR "juvenility"[All Fields] OR "paediatic*"[All Fields] OR "pediatric*"[All Fields] OR "child*"[All Fields] OR "infant*"[All Fields] OR "Child"[MeSH Terms] OR "Pediatrics"[MeSH Terms] OR "Infant"[MeSH Terms]) AND ("Uveitis"[MeSH Terms] OR "Uveitis"[All Fields] OR "uveitides"[All Fields] OR "uveitic"[All Fields] OR "uveit*"[All Fields] OR "Uveitis"[MeSH Terms]) AND ("intraocular lens"[All Fields] OR "IOL"[All Fields] OR "lenses, intraocular"[MeSH Terms]) AND ("aphake"[All Fields] OR "aphakes"[All Fields] OR "aphakic"[All Fields] OR "aphakics"[All Fields] OR ("Aphakia"[MeSH Terms] OR "Aphakia"[All Fields] OR "aphakias"[All Fields]) OR "aphak*"[All Fields] OR "Aphakia"[MeSH Terms])<br>**Translations**<br>**juvenile:** "juvenile"[All Fields] OR "juvenile's"[All Fields] OR "juveniles"[All Fields] OR "juvenility"[All Fields]<br>**uveitis:** "uveitis"[MeSH Terms] OR "uveitis"[All Fields] OR "uveitides"[All Fields]<br>**aphakic:** "aphake"[All Fields] OR "aphakes"[All Fields] OR "aphakic"[All Fields] OR "aphakics"[All Fields]<br>**aphakia:** "aphakia"[MeSH Terms] OR "aphakia"[All Fields] OR "aphakias"[All Fields]	45	14:40:21

Showing 1 to 1 of 1 entries

Cochrane Central:

Search Name:
Date Run: 12/11/2020 19:52:49
Comment:

ID	Search	Hits
#1	juvenile	3896
#2	paediatic*	11
#3	pediatric*	60911
#4	child*	177348
#5	infant*	63392
#6	MeSH descriptor: [Child] explode all trees	55654
#7	MeSH descriptor: [Pediatrics] explode all trees	674
#8	MeSH descriptor: [Infant] explode all trees	31920
#9	uveitis	1369
#10	uveitic	137
#11	uveit*	1412
#12	MeSH descriptor: [Uveitis] explode all trees	607
#13	"intraocular lens"	2240
#14	IOL	2210
#15	MeSH descriptor: [Lenses, Intraocular] explode all trees	998
#16	aphakic	215
#17	aphakia	231
#18	aphak*	354
#19	MeSH descriptor: [Aphakia] explode all trees	97
#20	(#1 OR #2 OR #3 OR #4 OR #5 OR #6 OR #7 OR #8) AND (#9 OR #10 OR #11 OR #12) AND (#13 OR #14 OR #15) AND (#16 OR #17 OR #18 OR #19)	3

EMBASE:

Search History
(24searches found)

Search history sorted by search number ascending

# ▲	Searches	Results	Type	Actions	Annota
1	juvenile.mp. [mp=title, abstract, heading word, drug trade name, original title, device manufacturer, drug manufacturer, device trade name, keyword, floating subheading word, candidate term word]	144788	Advanced	Display Results More	
2	paediatic*.mp. [mp=title, abstract, heading word, drug trade name, original title, device manufacturer, drug manufacturer, device trade name, keyword, floating subheading word, candidate term word]	45	Advanced	Display Results More	
3	pediatric*.mp. [mp=title, abstract, heading word, drug trade name, original title, device manufacturer, drug manufacturer, device trade name, keyword, floating subheading word, candidate term word]	565766	Advanced	Display Results More	
4	child*.mp. [mp=title, abstract, heading word, drug trade name, original title, device manufacturer, drug manufacturer, device trade name, keyword, floating subheading word, candidate term word]	2730268	Advanced	Display Results More	
5	infant*.mp. [mp=title, abstract, heading word, drug trade name, original title, device manufacturer, drug manufacturer, device trade name, keyword, floating subheading word, candidate term word]	883201	Advanced	Display Results More	
6	exp child/	2666476	Advanced	Display Results More	

7	exp pediatrics/	108982	Advanced	Display Results More
8	exp infant/	1001185	Advanced	Display Results More
9	uveitis.mp. [mp=title, abstract, heading word, drug trade name, original title, device manufacturer, drug manufacturer, device trade name, keyword, floating subheading word, candidate term word]	35116	Advanced	Display Results More
10	uveitic.mp. [mp=title, abstract, heading word, drug trade name, original title, device manufacturer, drug manufacturer, device trade name, keyword, floating subheading word, candidate term word]	1402	Advanced	Display Results More
11	uveit*.mp. [mp=title, abstract, heading word, drug trade name, original title, device manufacturer, drug manufacturer, device trade name, keyword, floating subheading word, candidate term word]	35491	Advanced	Display Results More
12	exp uveitis/	55715	Advanced	Display Results More
13	"intraocular lens".mp. [mp=title, abstract, heading word, drug trade name, original title, device manufacturer, drug manufacturer, device trade name, keyword, floating subheading word, candidate term word]	18889	Advanced	Display Results More
14	IOL.mp. [mp=title, abstract, heading word, drug trade name, original title, device manufacturer, drug manufacturer, device trade name, keyword, floating subheading word, candidate term word]	13728	Advanced	Display Results More
15	exp lens implant/	24928	Advanced	Display Results More

16	aphakic.mp. [mp=title, abstract, heading word, drug trade name, original title, device manufacturer, drug manufacturer, device trade name, keyword, floating subheading word, candidate term word]	3214	Advanced	Display Results More	
17	aphakia.mp. [mp=title, abstract, heading word, drug trade name, original title, device manufacturer, drug manufacturer, device trade name, keyword, floating subheading word, candidate term word]	5481	Advanced	Display Results More	
18	aphak*.mp. [mp=title, abstract, heading word, drug trade name, original title, device manufacturer, drug manufacturer, device trade name, keyword, floating subheading word, candidate term word]	6806	Advanced	Display Results More	
19	exp aphakia/	4782	Advanced	Display Results More	
20	1 or 2 or 3 or 4 or 5 or 6 or 7 or 8	3543982	Advanced	Display Results More	
21	9 or 10 or 11 or 12	59370	Advanced	Display Results More	
22	13 or 14 or 15	32334	Advanced	Display Results More	
23	16 or 17 or 18 or 19	6806	Advanced	Display Results More	
24	20 and 21 and 22 and 23	72	Advanced	Display Results More	

ClinicalTrials.gov:

5 Studies found for: **intraocular lens | Uveitis | Child**

Applied Filters: **Child (birth–17)**

ClinicalTrials.gov Search Results 11/12/2020

	Title	Status	Study Results	Conditions	Interventions	Locations
1	Retisert and Cataract Surgery in Patients With Severe Uveitis	Completed	No Results Available	•Intermediate Uveitis<br>•Posterior Uveitis	•Device: Retisert (fluocinolone acetonide implant)	•Duke University Medical Center, Durham, North Carolina, United States
2	Modified Intraocular Lens to Reduce Eye Inflammation After Cataract Surgery in Uveitis Patients	Completed	No Results Available	•Cataracts	•Device: heparin-surface modified intraocular lens	•National Eye Institute (NEI), Bethesda, Maryland, United States
3	Feasibility Study and Preliminary Application Study on Iris OCTA	Unknown status	No Results Available	•Conjunctivitis<br>•Glaucoma<br>•Myopia<br>•Diabetic Retinopathy<br>•Retinal Detachment<br>•Retinal Neovascularization, Unspecified<br>•Uveitis	•Diagnostic Test: OCTA (ZEISS)	
4	Uveitis/Intraocular Inflammatory Disease Biobank (iBank)	Recruiting	No Results Available	•Uveitis		•National Institutes of Health Clinical Center, 9000 Rockville Pike, Bethesda, Maryland, United States
5	Loteprednol vs Prednisolone for the Treatment of Intraocular Inflammation Following Cataract Surgery in Children.	Completed	Has Results	•Cataract	•Drug: Loteprednol etabonate<br>•Drug: Prednisolones acetate	•Bausch & Lomb Inc, Rochester, New York, United States

CINAHL:

Search History/Alerts

- Print Search History
  - Retrieve Searches
  - Retrieve Alerts
  - Save Searches / Alerts

Select / deselect all

Search ID#	**Search Terms**	**Search Options**	**Actions**
S1	(juvenile OR paediatric* OR pediatric* OR child* OR infant*) AND (uveitis OR uveitic OR uveit*) AND ("intraocular lens" OR IOL) AND (aphakic OR aphakia OR aphak*)	**Expanders** - Apply equivalent subjects **Search modes** - Proximity within five words	View Results (2) View Details **Edit**

- Relevance

Combined search into following databases: Web of Science (WOS), BIOSIS Previews (BIOSIS), Current Contents Connect (CCC), Data Citation Index (DRCI), Derwent Innovations Index (DIIDW), KCI-Korean Journal Database (KJD), Russian Science Citation Index (RSCI), SciELO Citation Index (SCIELO):

Results: 58

(from All Databases)

You searched for: **TOPIC:**((juvenile OR paediatric* OR pediatric* OR child* OR infant*) AND (uveitis OR uveitic OR uveit*) AND ("intraocular lens" OR IOL) AND (aphakic OR aphakia OR aphak*))

Timespan: All years. **Databases:** WOS, BIOSIS, CCC, DRCI, DIIDW, KJD, MEDLINE, RSCI, SCIELO, ZOOREC.

Search language=Auto

Supplementary file 2. Details of the meta-analysis on the pre-operative best-corrected visual acuity, including Funnel plot and sensitivity analysis.

Meta-analysis

Study	WMD	LCI 95%	HCI 95%	weight (%)
Guindolet et al. 2014	-0,67	-1,08	-0,26	16,83
Kotaniemi & Penttilä 2006	-0,59	-1,90	0,72	4,68
Yangzes et al. 2019	-0,49	-0,83	-0,15	18,42
Quinones et al. 2009	-0,23	-0,58	0,12	18,22
Sijssens et al. 2010	-0,22	-0,49	0,05	19,88
Kemp et al. 2015	-0,13	-1,39	1,13	4,98
BenEzra & Cohen 2000	0,53	0,13	0,93	16,99
Pooled	-0,23	-0,55	0,08	100,00
Statistics				
I-squared	71,36	37,73	86,82	
Cochran's Q	20,95			
Chi2, p	0,00			
tau2	0,11			

Funnel plot

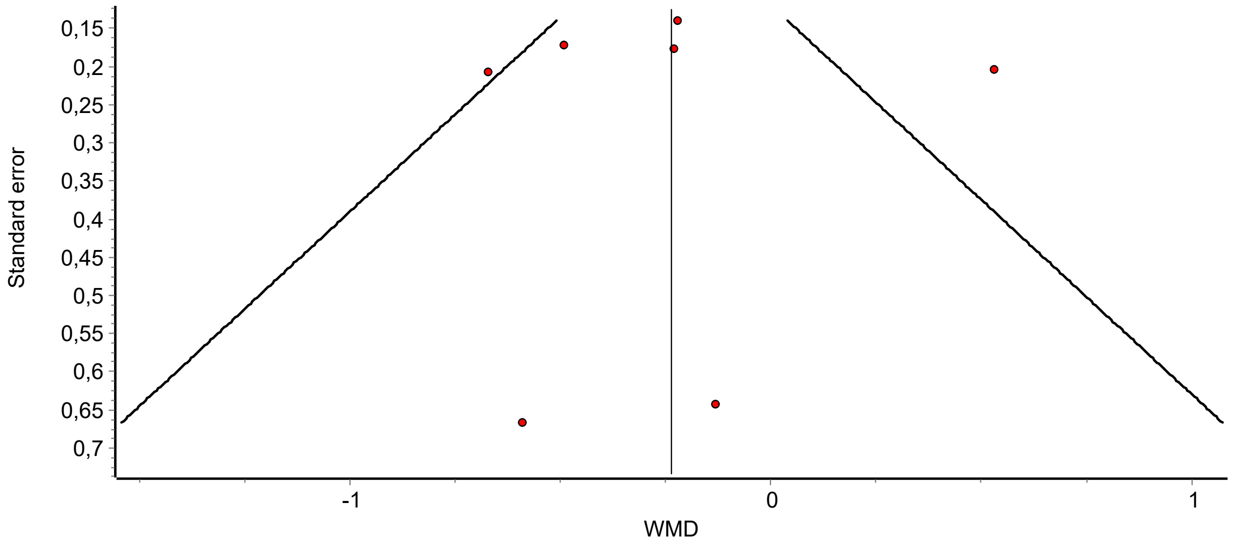

Sensitivity analysis

Excluded study	Pooled WMD	LCI 95%	HCI 95%	Cochran Q	p	I 2	I 2 LCI 95%	I 2 HCI 95%
Guindolet et al. 2014	-0,14	-0,48	0,19	15,85	0,01	68,45	25,45	86,64
Kotaniemi & Penttilä 2006	-0,21	-0,55	0,12	20,66	0,00	75,80	45,53	89,25
Yangzes et al. 2019	-0,18	-0,55	0,20	18,21	0,00	72,54	36,72	88,08
Quinones et al. 2009	-0,24	-0,63	0,16	20,95	0,00	76,13	46,42	89,37
Sijssens et al. 2010	-0,24	-0,66	0,18	20,93	0,00	76,11	46,37	89,36
Kemp et al. 2015	-0,24	-0,57	0,10	20,92	0,00	76,10	46,34	89,35
BenEzra & Cohen 2000	-0,36	-0,52	-0,20	4,59	0,47	0,00	0,00	72,37

Re-analysis after exclusion of BenEzra & Cohen (2000):

Meta-analysis

Study	WMD	LCI 95%	HCI 95%	weight (%)
Guindolet et al. 2014	-0,67	-1,08	-0,26	16,01
Kotaniemi & Penttilä 2006	-0,59	-1,90	0,72	1,55
Yangzes et al. 2019	-0,49	-0,83	-0,15	23,20
Quinones et al. 2009	-0,23	-0,58	0,12	22,01
Sijssens et al. 2010	-0,22	-0,49	0,05	35,56
Kemp et al. 2015	-0,13	-1,39	1,13	1,68
Pooled	-0,36	-0,52	-0,20	100,00
Statistics				
I-squared	0,00	0,00	72,37	
Cochran's Q	4,59			
Chi2, p	0,47			
tau2	0,00			

Forest plot

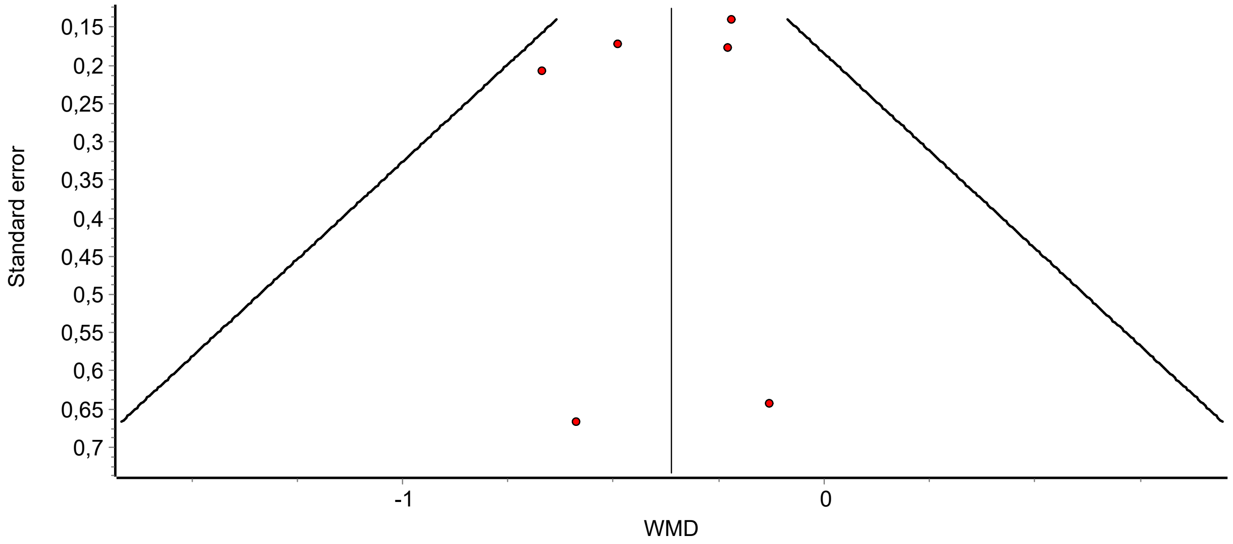

Sensitivity analysis

Excluded study	Pooled WMD	LCI 95%	HCI 95%	Cochran Q	p	I 2	I 2 LCI 95%	I 2 HCI 95%
Guindolet et al. 2014	-0,30	-0,48	-0,12	1,96	0,74	0,00	0,00	57,50
Kotaniemi & Penttilä 2006	-0,36	-0,54	-0,19	4,47	0,35	10,56	0,00	81,40
Yangzes et al. 2019	-0,32	-0,51	-0,14	3,86	0,42	0,00	0,00	78,48
Quinones et al. 2009	-0,40	-0,58	-0,21	3,89	0,42	0,00	0,00	78,61
Sijssens et al. 2010	-0,44	-0,64	-0,24	3,00	0,56	0,00	0,00	72,28
Kemp et al. 2015	-0,37	-0,55	-0,19	4,46	0,35	10,31	0,00	81,35

Supplementary file 3. Details of the meta-analysis on the post-operative short-term best-corrected visual acuity, including Funnel plot and sensitivity analysis.

Meta-analysis

Study	WMD	LCI 95%	HCI 95%	weight (%)
Kotaniemi & Penttilä 2006	-1,77	-3,45	-0,09	1,40
Yangzes et al. 2019	-0,51	-0,86	-0,16	20,76
Quinones et al. 2009	-0,26	-0,58	0,06	22,82
Sijssens et al. 2010	-0,15	-0,38	0,08	32,13
Kemp et al. 2015	-0,03	-0,94	0,88	4,44
Guindolet et al. 2014	0,01	-0,64	0,66	8,10
BenEzra & Cohen 2000	0,11	-0,45	0,67	10,35
Pooled	-0,23	-0,43	-0,03	100,00
Statistics				
I-squared	28,08	0,00	68,99	
Cochran's Q	8,34			
Chi2, p	0,21			
tau2	0,02			

Funnel plot

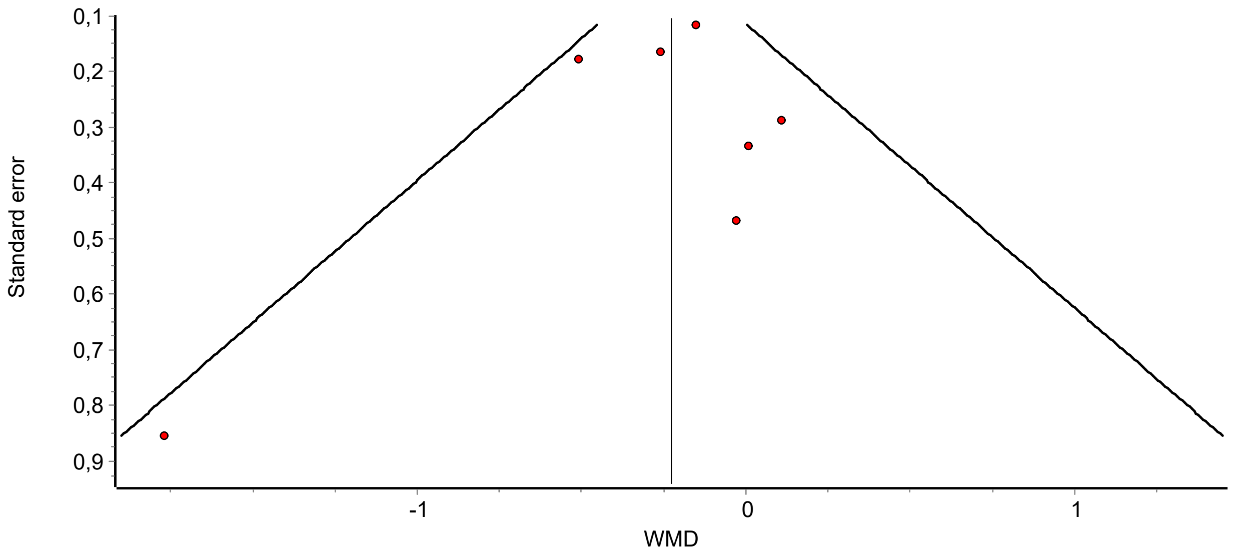

Sensitivity analysis:

Excluded study	Pooled WMD	LCI 95%	HCI 95%	Cochran Q	p	I 2	I 2 LCI 95%	I 2 HCI 95%
Kotaniemi & Penttilä 2006	-0,21	-0,36	-0,06	5,05	0,41	0,91	0,00	74,85
Yangzes et al. 2019	-0,16	-0,33	0,02	5,15	0,40	2,91	0,00	75,36
Quinones et al. 2009	-0,22	-0,49	0,05	8,28	0,14	39,63	0,00	76,05
Sijssens et al. 2010	-0,26	-0,53	0,02	7,63	0,18	34,44	0,00	73,71
Kemp et al. 2015	-0,24	-0,46	-0,01	8,17	0,15	38,76	0,00	75,68
Guindolet et al. 2014	-0,25	-0,47	-0,03	7,82	0,17	36,06	0,00	74,47
BenEzra & Cohen 2000	-0,27	-0,47	-0,06	6,88	0,23	27,37	0,00	69,94

Supplementary file 4. Details of the meta-analysis on the post-operative long-term best-corrected visual acuity, including Funnel plot and sensitivity analysis.

Meta-analysis

Study	WMD	LCI 95%	HCI 95%	weight (%)
Kotaniemi & Penttilä 2006	-1,77	-3,45	-0,09	0,97
Yangzes et al. 2019	-0,51	-0,86	-0,16	22,50
Sijssens et al. 2010	-0,30	-0,58	-0,02	34,33
Quinones et al. 2009	-0,37	-0,67	-0,07	30,39
Kemp et al. 2015	-0,03	-1,05	0,99	2,64
BenEzra & Cohen 2000	0,00	-0,99	0,99	2,77
Guindolet et al. 2014	0,01	-0,64	0,66	6,40
Pooled	-0,35	-0,51	-0,18	100,00
Statistics				
I-squared	0,00	0,00	69,51	
Cochran's Q	5,74			
Chi2, p	0,45			
tau2	0,00			

Forest plot

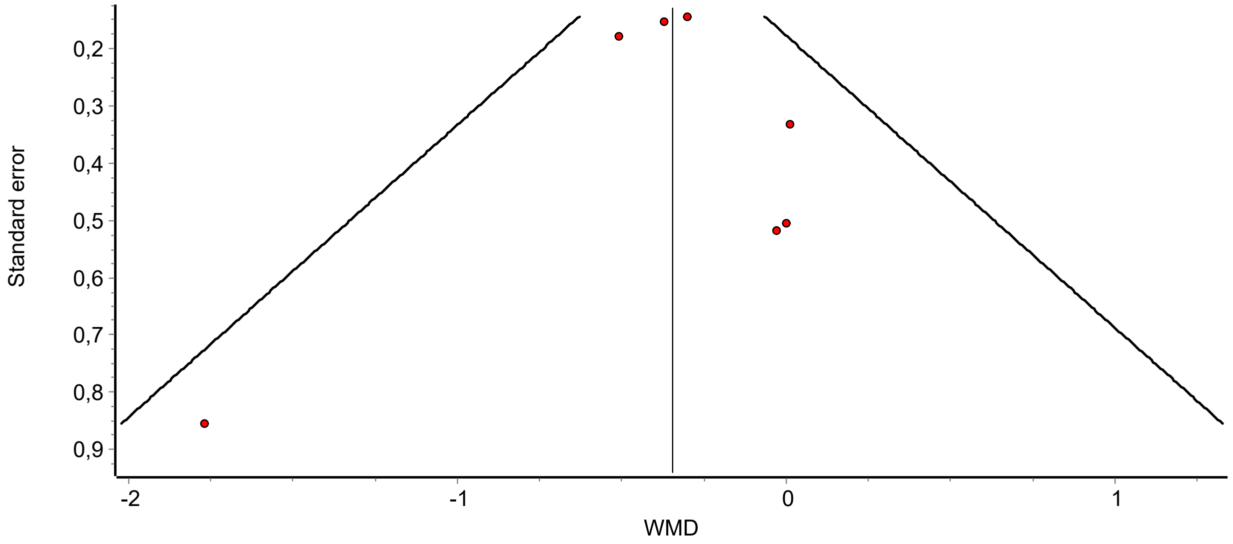

Sensitivity analysis

Excluded study	Pooled WMD	LCI 95%	HCI 95%	Cochran Q	p	I 2	I 2 LCI 95%	I 2 HCI 95%
Kotaniemi & Penttilä 2006	-0,33	-0,50	-0,17	2,95	0,71	0,00	0,00	56,95
Yangzes et al. 2019	-0,30	-0,49	-0,11	4,66	0,46	0,00	0,00	72,78
Sijssens et al. 2010	-0,36	-0,59	-0,13	5,58	0,35	10,36	0,00	77,25
Quinones et al. 2009	-0,33	-0,56	-0,10	5,71	0,34	12,47	0,00	77,79
Kemp et al. 2015	-0,36	-0,53	-0,18	5,36	0,37	6,68	0,00	76,32
BenEzra & Cohen 2000	-0,36	-0,53	-0,18	5,26	0,39	4,89	0,00	75,87
Guindolet et al. 2014	-0,37	-0,54	-0,20	4,51	0,48	0,00	0,00	71,86

Supplementary file 5. Details of the meta-analysis on the post-operative cystoid macular edema, including Funnel plot and sensitivity analysis.

Meta-analysis

Study	OR	LCI 95%	HCI 95%	weight (%)
BenEzra & Cohen 2000	9,80	0,44	219,25	15,17
Guindolet et al. 2014	7,53	0,35	160,86	15,45
Kotaniemi & Penttilä 2006	0,11	0,01	2,39	15,63
Sijssens et al. 2010	0,14	0,02	1,35	21,51
Yangzes et al. 2019	0,45	0,14	1,44	32,24
Pooled	0,70	0,15	3,29	100,00
Statistics				
I-squared	53,61	0,00	82,92	
Cochran's Q	8,62			
Chi2, p	0,07			
tau2	1,57			

Forest plot

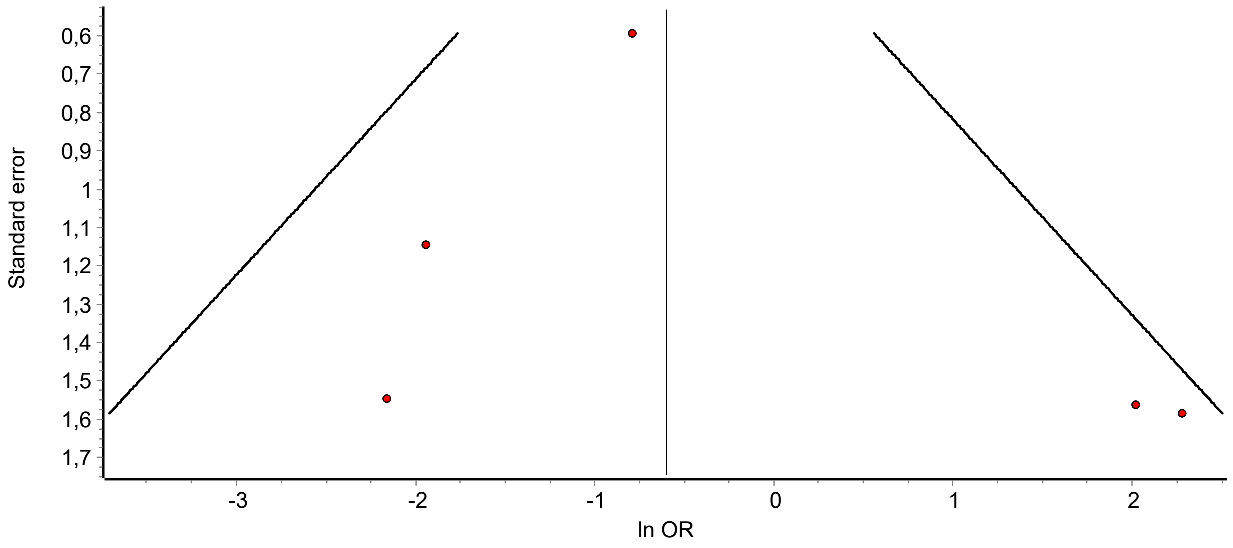

Sensitivity analysis

Excluded study	Pooled OR	LCI 95%	HCI 95%	Cochran Q	p	I 2	I 2 LCI 95%	I 2 HCI 95%
BenEzra & Cohen 2000	0,43	0,10	1,76	5,02	0,17	40,20	0,00	79,73
Guindolet et al. 2014	0,45	0,10	2,01	5,55	0,14	45,93	0,00	82,01
Kotaniemi & Penttilä 2006	1,02	0,17	6,03	7,51	0,06	60,06	0,00	86,66
Sijssens et al. 2010	1,13	0,17	7,39	6,99	0,07	57,06	0,00	85,75
Yangzes et al. 2019	0,93	0,09	9,97	8,37	0,04	64,16	0,00	87,88

Supplementary file 6. Details of the meta-analysis on the post-operative glaucoma, including Funnel plot and sensitivity analysis.

Study	OR	LCI 95%	HCI 95%	weight (%)
Artigas et al. 2017	2,69	0,10	73,20	4,91
BenEzra & Cohen 2000	3,32	0,12	91,60	4,86
Kotaniemi & Penttilä 2006	7,00	0,34	145,24	5,83
O'Rourke et al. 2000	0,41	0,01	12,64	4,54
Sijssens et al. 2010	0,90	0,28	2,88	39,24
Yangzes et al. 2019	2,02	0,64	6,36	40,61
Pooled	1,52	0,73	3,17	100,00
Statistics				
I-squared	0,00	0,00	56,07	
Cochran's Q	2,89			
Chi2, p	0,72			
tau2	0,00			

Forest plot

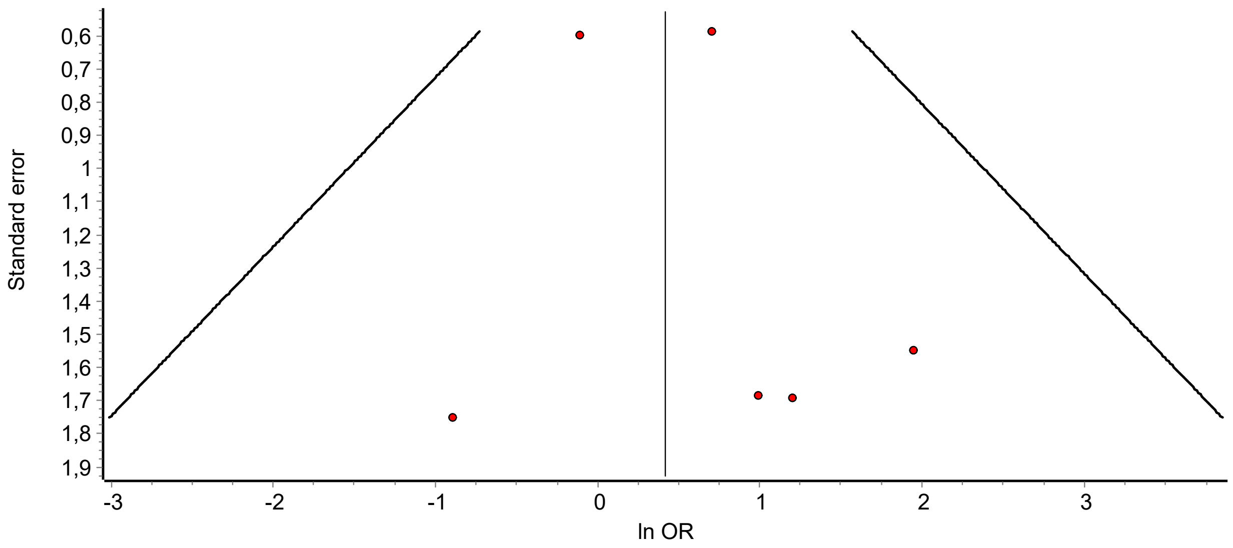

Sensitivity analysis

Excluded study	Pooled OR	LCI 95%	HCI 95%	Cochran Q	p	I 2	I 2 LCI 95%	I 2 HCI 95%
Artigas et al. 2017	1,48	0,70	3,13	2,77	0,60	0,00	0,00	69,95
BenEzra & Cohen 2000	1,46	0,69	3,10	2,67	0,62	0,00	0,00	68,80
Kotaniemi & Penttilä 2006	1,39	0,65	2,95	1,86	0,76	0,00	0,00	55,19
O'Rourke et al. 2000	1,62	0,77	3,43	2,29	0,68	0,00	0,00	63,75
Sijssens et al. 2010	2,15	0,84	5,49	1,58	0,81	0,00	0,00	47,30
Yangzes et al. 2019	1,26	0,49	3,25	2,50	0,64	0,00	0,00	66,75

Supplementary file 7. Details of the meta-analysis on the post-operative visual axis opacification, including Funnel plot and sensitivity analysis.

Meta-analysis

Study	OR	LCI 95%	HCI 95%	weight (%)
Artigas et al. 2017	15,00	0,52	430,47	7,33
BenEzra & Cohen 2000	14,54	0,67	316,69	8,70
Guindolet et al. 2014	1,44	0,05	40,54	7,43
Kemp et al. 2015	7,00	0,25	192,26	7,52
Kotaniemi & Penttilä 2006	5,63	0,27	116,99	8,97
ORourke et al. 2000	0,27	0,01	8,46	7,00
Yangzes et al. 2019	10,40	2,99	36,21	53,05
Pooled	6,76	2,73	16,78	100,00
Statistics				
I-squared	0,00	0,00	65,69	
Cochran's Q	5,11			
Chi2, p	0,53			
tau2	0,00			

Forest plot

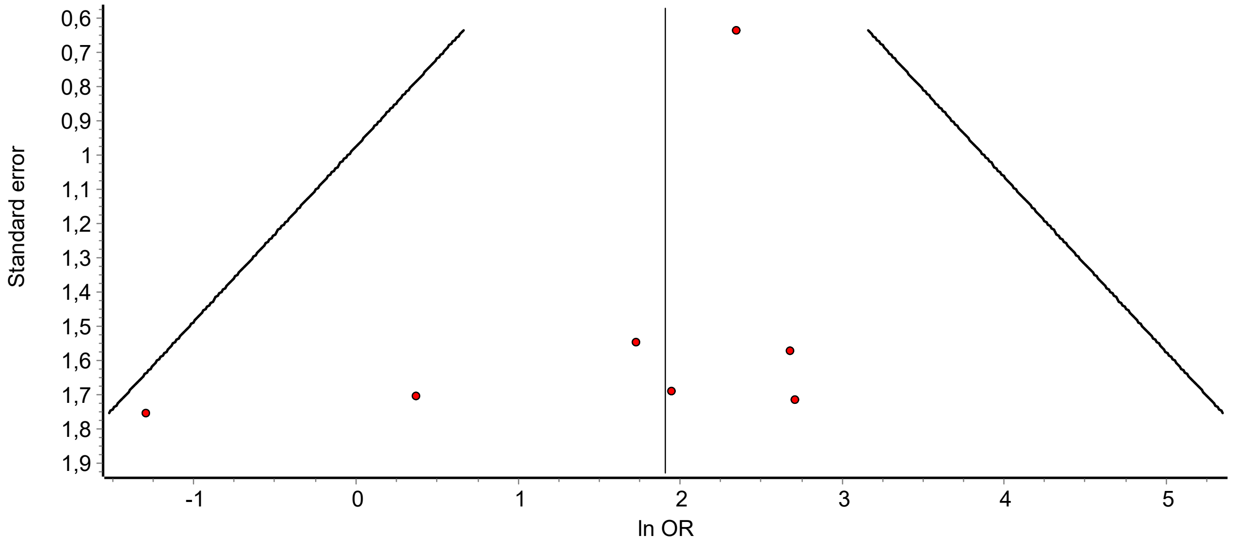

Sensitivity analysis

Excluded study	Pooled OR	LCI 95%	HCI 95%	Cochran Q	p	I 2	I 2 LCI 95%	I 2 HCI 95%
Artigas et al. 2017	6,35	2,47	16,32	4,87	0,43	0,00	0,00	73,96
BenEzra & Cohen 2000	6,29	2,43	16,27	4,85	0,43	0,00	0,00	73,82
Guindolet et al. 2014	7,66	2,98	19,68	4,22	0,52	0,00	0,00	69,90
Kemp et al. 2015	6,61	2,50	17,49	5,10	0,40	2,05	0,00	75,15
Kotaniemi & Penttilä 2006	6,77	2,55	17,99	5,09	0,40	1,77	0,00	75,07
ORourke et al. 2000	8,61	3,36	22,10	1,50	0,91	0,00	0,00	15,15
Yangzes et al. 2019	4,16	1,10	15,67	4,13	0,53	0,00	0,00	69,29

Supplementary file 8. Details of the meta-analysis on the post-operative hypotony, including Funnel plot and sensitivity analysis.

Meta-analysis

Study	OR	LCI 95%	HCI 95%	weight (%)
BenEzra & Cohen 2000	0,30	0,01	8,33	23,66
O'Rourke et al. 2000	0,53	0,01	20,19	19,66
Sijssens et al. 2010	0,06	0,00	1,16	29,23
Yangzes et al. 2019	0,21	0,01	4,67	27,46
Pooled	0,19	0,04	0,95	100,00
Statistics				
I-squared	0,00	0,00	53,35	
Cochran's Q	0,98			
Chi2, p	0,80			
tau2	0,00			

Forest plot

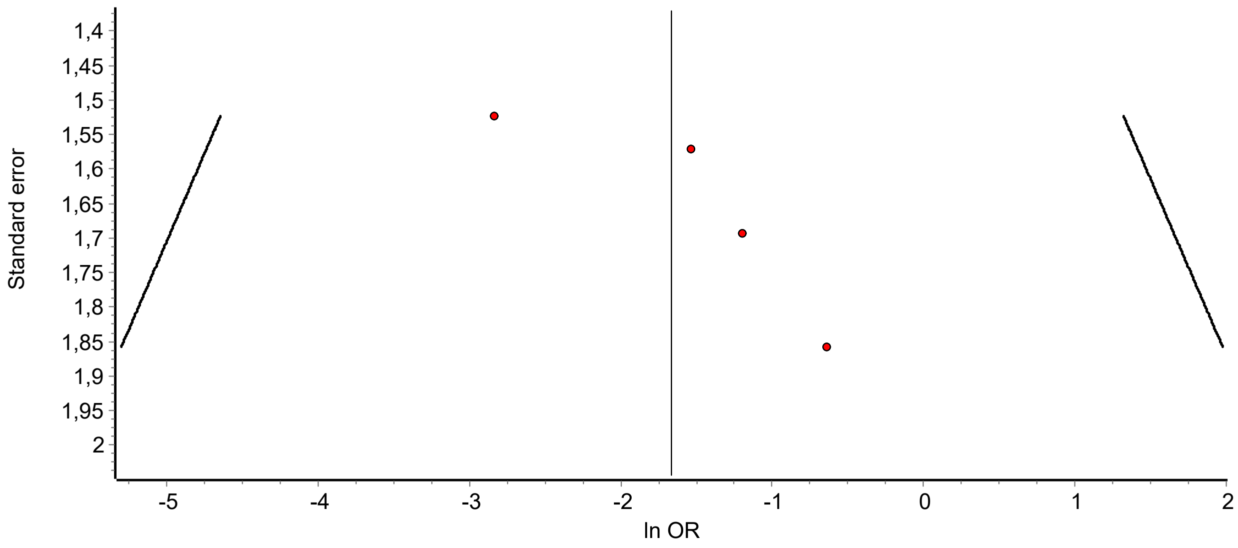

Sensitivity analysis

Excluded study	Pooled OR	LCI 95%	HCI 95%	Cochran Q	p	I 2	I 2 LCI 95%	I 2 HCI 95%
BenEzra & Cohen 2000	0,16	0,03	1,04	0,89	0,64	0,00	0,00	76,54
O'Rourke et al. 2000	0,15	0,02	0,89	0,61	0,74	0,00	0,00	65,64
Sijssens et al. 2010	0,31	0,05	2,10	0,14	0,93	0,00	0,00	0,00
Yangzes et al. 2019	0,18	0,03	1,21	0,98	0,61	0,00	0,00	78,69

Supplementary file 9. Details of the meta-analysis on the post-operative posterior synechia, including Funnel plot and sensitivity analysis.

Meta-analysis

Study	OR	LCI 95%	HCI 95%	weight (%)
BenEzra & Cohen 2000	14,54	0,67	316,69	33,93
Guindolet et al. 2014	7,53	0,35	160,86	34,23
Kemp et al. 2015	0,40	0,02	10,02	31,84
Pooled	3,70	0,44	31,11	100,00
Statistics				
I-squared	28,49	0,00	92,56	
Cochran's Q	2,80			
Chi2, p	0,25			
tau2	1,01			

Forest plot

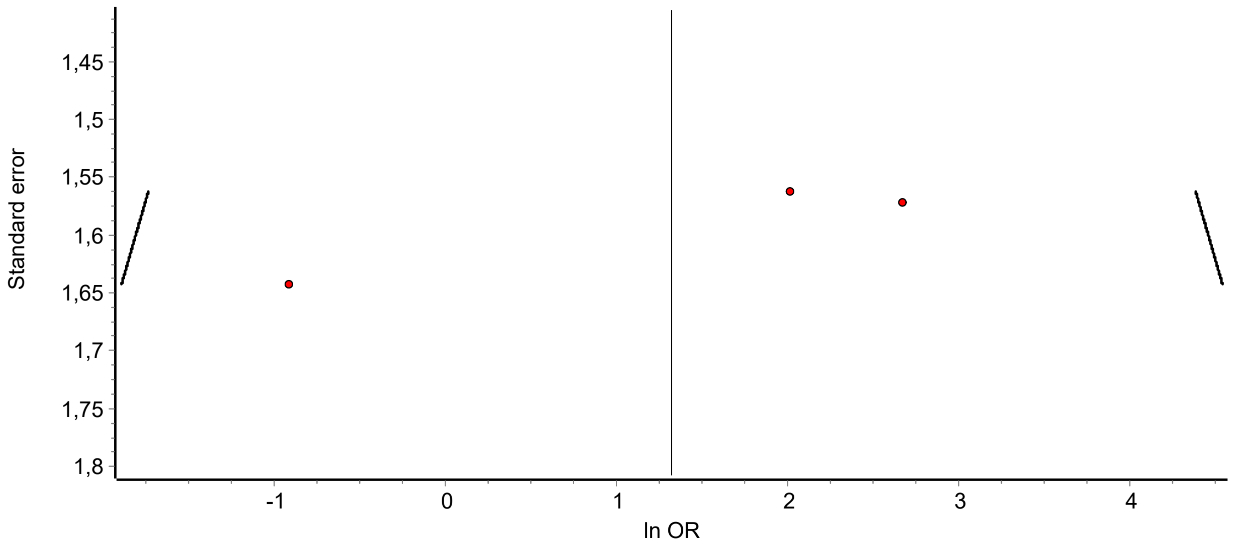

Sensitivity analysis

Excluded study	Pooled OR	LCI 95%	HCI 95%	Cochran Q	p	I 2	I 2 LCI 95%	I 2 HCI 95%
BenEzra & Cohen 2000	1,81	0,10	32,13	1,68	0,20	40,31	NAN	NAN
Guindolet et al. 2014	2,49	0,07	84,16	2,50	0,11	59,94	0,00	90,63
Kemp et al. 2015	10,44	1,19	91,61	0,09	0,77	0,00	NAN	NAN

Supplementary file 10. Details of the meta-analysis on the post-operative retinal detachment, including Funnel plot and sensitivity analysis.

Meta-analysis

Study	OR	LCI 95%	HCI 95%	weight (%)
BenEzra & Cohen 2000	1,00	0,02	55,27	14,10
Guindolet et al. 2014	1,44	0,05	40,54	20,42
Kotaniemi & Penttilä 2006	0,30	0,01	8,72	19,83
ORourke et al. 2000	0,53	0,01	20,19	17,13
Yangzes et al. 2019	1,15	0,07	19,38	28,53
Pooled	0,79	0,18	3,57	100,00
Statistics				
I-squared	0,00	0,00	0,00	
Cochran's Q	0,58			
Chi2, p	0,97			
tau2	0,00			

Forest plot

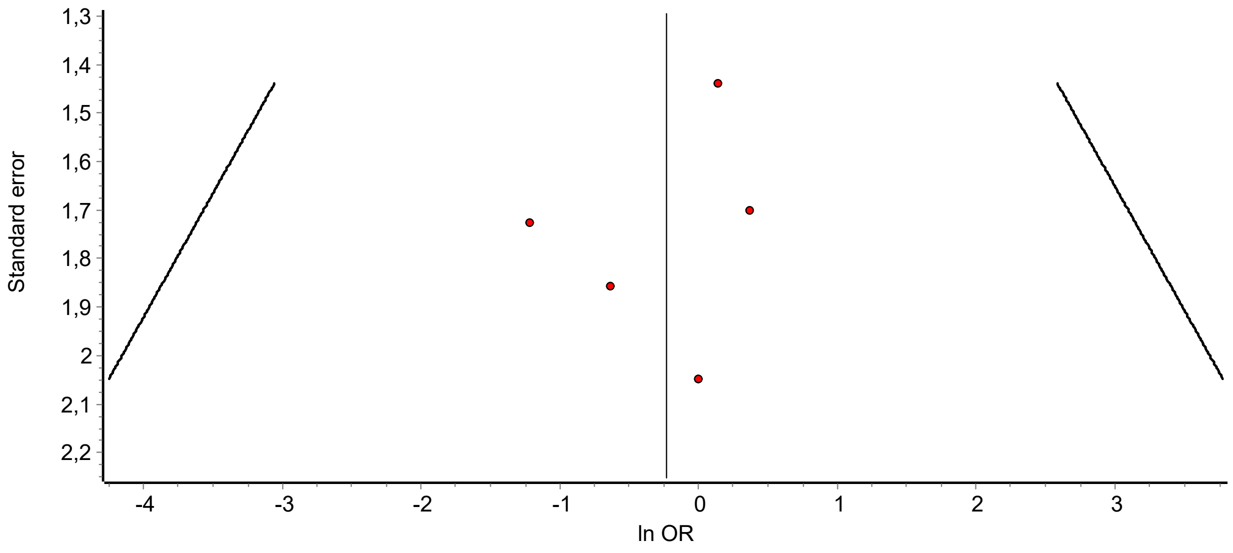

Sensitivity analysis

Excluded study	Pooled OR	LCI 95%	HCI 95%	Cochran Q	p	I 2	I 2 LCI 95%	I 2 HCI 95%
BenEzra & Cohen 2000	0,76	0,15	3,87	0,56	0,90	0,00	0,00	18,45
Guindolet et al. 2014	0,68	0,13	3,67	0,42	0,94	0,00	0,00	0,00
Kotaniemi & Penttilä 2006	1,01	0,19	5,43	0,17	0,98	0,00	0,00	0,00
ORourke et al. 2000	0,86	0,16	4,50	0,52	0,91	0,00	0,00	12,04
Yangzes et al. 2019	0,68	0,11	4,04	0,48	0,92	0,00	0,00	4,75
